# Theoretical studies of optoelectronic, magnetization and heat transport properties of conductive metal adatoms adsorbed on edge chlorinated nanographenes†

**DOI:** 10.1039/c8ra02032a

**Published:** 2018-05-15

**Authors:** Ruby Srivastava

**Affiliations:** Center for Molecular Modeling, CSIR-Indian Institute of Chemical Technology Hyderabad-500607 India amitruby1@gmail.com

## Abstract

The electronic structures, magnetization and quantum transport properties of edge chlorinated nanographenes (Cl NGRs) (C1–C3) functionalized with conductive metal adatoms (Al, Au and Cu) has been investigated by means of density functional theory (DFT) with periodic boundary conditions and plane wave basis functions. The adsorption energy results depict weak chemisorption and strong physisorption for Au adsorption for C1, while C2 and C3 show strong chemisorption towards the studied metals. The role of dispersion forces has also been studied with an empirical classical model. The results show that the metal clusters avoid hollow sites on the Cl NGRs surface and favor atop and bond sites. The net magnetic moment of 0.73 *μ*_B_ is observed for the (Cl NGRs–metals) system and is in reasonable agreement with the previous calculations carried out on graphene nanoribbons. The TDDFT calculations predict that the absorption spectra for metal dimer–Cl NGRs lie in the visible region. The predictive electrical conductivity of these systems suggests that the metal adatoms play an important role in the transport properties of devices and can be used for thermoelectric applications.

## Abstract 2

The electronic structures, magnetization and quantum transport properties of edge chlorinated nanographenes functionalized with conductive metal adatoms have been investigated by means of density functional theory with plane wave basis functions.

## Graphical abstract

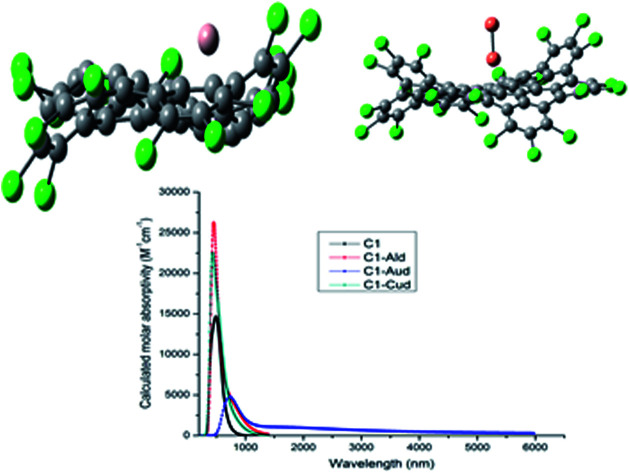

## 1. Introduction

Graphene, a monolayer of carbon atoms, arranged in honeycomb network has attracted immense interest^1–3^ due to its optoelectronic, transport^3–8^ and extraordinary physical properties, which is governed by the size of the sheet and the nature of its periphery.^9,10^ Graphene is a zero-gap semiconductor with a point-like Fermi surface and a linear dispersion at the Fermi level. These characteristics of graphene are responsible for the observed anomalous quantum Hall effect, the massless Dirac electrons and the high charge-carrier mobility.^11,12^ The physico-chemical properties of graphene can be modified by chemical functionalization, followed by two approaches.^12–14^ The first one is functionalization on the basal plane of graphene *via* covalent addition to C=C bonds and the other one is at the edge *via* replacement of peripheral hydrogen atoms with functional groups. The edges of graphene are chemically active and prone to structural modifications and interactions with the gas dissolved in the environment, thereby affecting the properties of graphene as well. In comparison to pristine nanographenes, these functionalized molecules show characteristic non-planar molecular geometry, which strongly infiuence their crystal packing. Both edge-chlorinated nanographenes (Cl NGRs) and graphene nanoribbons (GNRs) exhibit enhanced solubility, defined and tunable band gaps, and decreased frontier molecular orbital energy levels.^15^ As the two-dimensional carbon material graphene is considered for sensors, electronics and catalysis applications due to its exceptional electric and mechanical properties, some of these applications require the adsorption of metal clusters onto graphene. Electronic properties that are intimately related to electron–electron interactions, *viz.*, the compressibility and plasmon dispersion in a two-dimensional electron gas show unique behavior in graphene. The compressibility of a two-dimensional electron gas is also an important physical quantity that is deduced from the ground state energy. It provides important information about the electron correlations, the chemical potential and the stability of the system, *etc.* Thus the metal–graphene systems have consequently become a subject of intense investigation. For computational studies of metal adsorption on graphene, *ab initio* methods such as density functional theory give the most accurate description of the metal–carbon interaction. As the *ab initio* methods are computationally expensive, so for simulations of larger systems, it is necessary to make use of interatomic model potentials. Among the existing model potentials for the interaction between metals and carbon, the Lennard-Jones potential, a simple pair potential modeling van der Waals (vdW) attraction is used for gold adsorbates. Computational studies indicate that the coinage metals adsorb more strongly to curved graphene surfaces than to flat graphene.^16,17^ The nature of the interaction between the surface and the cluster is also very interesting. The interaction between the cluster and the mirror induced dipole inside the surface is a possibility, but one should not ignore the role of van der Waals (dispersion) forces, which can be significant even in describing plain surfaces such as graphite *etc.* The modelling of dispersion forces, which arise from electron correlated effects, is demanding in the density functional theory because their effects are excluded from standard functionals. The adsorption of atoms and dimers of Ag and Au on graphite has also been studied previously with the DFT method, with a local density approximation (LDA).^18–21^ The interaction of transition metal atoms and their clusters with graphene can involve a large component of dispersion interactions, stemming from nonlocal electron–electron correlation. Theoretical studies of graphene and its complexes with transition elements have often involved density functional theory (DFT) utilizing local density approximation (LDA)^22^ or generalized-gradient approximation (GGA).^22–25^ However, these DFT approaches are based on a local approximation for the exchange–correlation functional, which cannot accurately represent the dispersion energy. Several techniques have been developed to improve these DFT approaches, which take account of dispersion interactions either explicitly or implicitly.^26,27^ The van der Waals density functional (vdW DF) method and its variations seem to be promising to describe the interaction of graphene with metals.^28,29^ A number of experimental and theoretical work has been carried out on electronic and magnetic behaviour of metal dimers and adatoms of different elements adsorbed on graphene system which yield many interesting results.^30–32^

Metallic nanowires have drawn significant attention due to the quantum confinement effect since they represent the ultimate miniaturization of conductors. Novel and interesting phenomena result in the nanoscales of metallic nanowires which is different from their bulk materials.^33–35^ The effect on quantized conductance has also been verified by numerous nanoscale systems experimentally,^32^ and the electronic structure, ballistic quantum transport properties, and e–pH coupling for atomic wires of Al and coinage metals has already been reported previously.^36^ A theoretical investigation for coinage metal atoms (Cu, Ag, Au) and clusters on graphite and graphene^36–38^ were carried out to observe the interaction, charge transfer, distances, nature of binding between graphene and metal, as well as the electronic and structural properties for understanding the metal–graphene interaction for the development of hybrid materials with specific properties. It has been observed that the conductivity of Al, Au and Cu atomic wires show opposite trends to those for bulk materials (example, Al > Cu > Au for atomic wires *vs.* Cu > Au > Al for bulk materials). Motivated by these studies, we have carried out the studies on the seven Cl NGRs structures (C_42_CI_18_ (C1), C_48_CI_18_ (C2) and C_60_Cl_22_ (C3), C4 (C_60_Cl_24_), C5 (C_96_Cl_27_H_3_), C6 (C_132_Cl_32_H_2_) and C7 (C_222_Cl_42_)) reported by Tan *et al.*,^15^ with conductive metal adatoms (Al, Au and Cu) (single and dimer) to investigate the adsorption energies, electronic, magnetic, absorption and transport properties of Cl NGRs–metal systems. For complexes C4–C7, we have reported the optimized structures of only reliable results in ESI.† The optimized results for C4, C5, C4–Au and C5–Au complexes are given in ESI Fig. 1.† The electronic and optical data of these structures are given in ESI Table 1 and ESI Table 2† respectively.

## 2. Computational details

The interaction of a C1, C2 and C3 molecule with a single and dimeric form of Al, Au and Cu atom is studied using (electronic) density functional theory. The DFT calculations were performed using the VASP^39^ with projector-augmented wave method (PAW) with PBE exchange correlation (XC) functional.^40^ The exchange and correction terms are described using generalized gradient approximation (GGA) in the scheme of the Perdew–Burke–Ernzerhof (PBE) functional. The plane-wave basis set included plane waves with an energy cut-off of 300 eV. Eigen values and eigenstates of the Kohn–Sham Hamiltonian have been calculated at *Γ* point only of a cubic cell of side (C1–Al-16.736 Å, C1–Au-16.961 Å, C1–Cu-20.748 Å, C1–Ald-19.792 Å, C1–Aud-17.592 Å, C1–Cud-16.491 Å), (C2–Al-20.081 Å, C2–Au-18.426 Å, C2–Cu-19.892 Å, C2–Ald-18.284 Å, C2–Aud-18.426 Å, C2–Cud-18.493 Å) and (C3–Al-23.081 Å, C3–Au-22.933 Å, C3–Cu-23.013 Å, C3–Ald-22.432 Å, C3–Aud-23.161 Å, C3–Cud-23.069 Å) with convergence threshold parameter of 1.00× × 10^−8^ for the optimized structures. In addition, the van der Waals density functional (vdW-DF) as proposed by Dion *et al.*^41^ is also used to calculate the energies of these models. To predict the accurate energies, the dipole–dipole correction and spin polarization effect is included in the calculations. The optimized geometries are further reoptimized with Gaussian package^42^ using PW91PW91/LANL2DZ^43^ basis set with (ECP) potential to calculate the Mulliken charges^44^ and HOMO–LUMO (HL) gap. The vibrational frequency analysis is carried out to investigate the stability of complexes. TDDFT calculations have been carried out to study the absorption spectra on metal dimers–Cl NGRs system. The ESP potential is also calculated for the five isolated (C_42_CI_18_ (C1), C_48_CI_18_ (C2) and C_60_Cl_22_ (C3), C_60_Cl_24_ (C4), C_96_Cl_27_H_3_ (C5)) complexes. The thermal conductivity is predictively calculated by RTIME = 52 femtosecond (approximation is taken from the previous report)^45^ and 3 ×× 3 ×× 3 *K*-points to get the desired results, by taking so many approximate values of RTIME and *K*-points combination.

## 3. Results

### 3.1 Optimized geometries of C1–C3

**Fig. 1:**
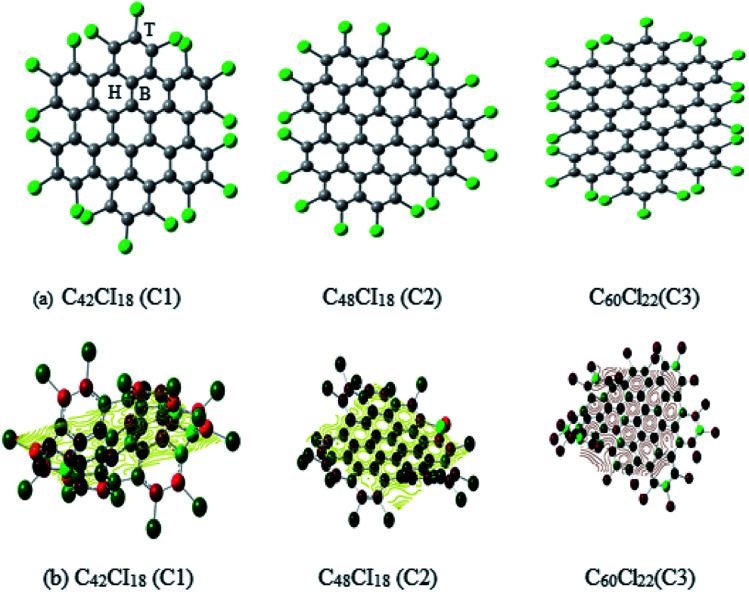
(a) The schematic representation of Cl NGRs (C1, C2 and C3), (b) spin polarized Cl NGRs with reported ESP potential C1 – −1773.65 au, C2 – −1861.16 au and C3 – −2295.06 au.

**Fig. 2:**
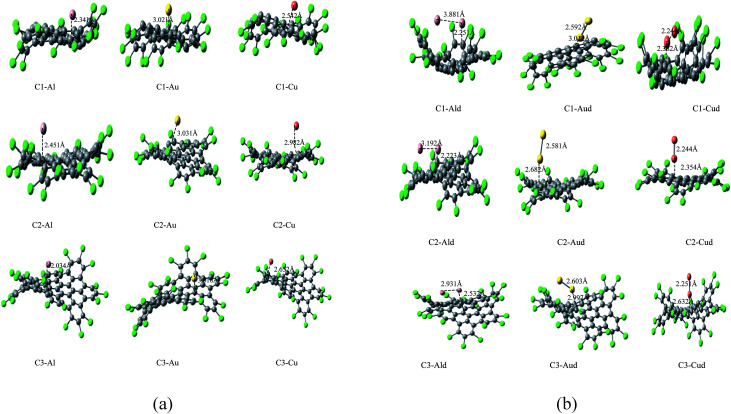
(a) Optimized geometries of C1–M, C2–M and C3–M (M = Al, Au and Cu) obtained by DFT calculations. (b) Optimized geometries of C1–Md, C2–Md and C3–Md (M = Al, Au and Cu) obtained by DFT calculations.

The structural representation of C1 (C_42_Cl_18_), C2 (C_48_Cl_18_), C3 (C_60_Cl_22_) is given in Fig. 1a and the ESP potential surface for the monomers are reported in Fig. 1b. The three possible sites for a single and dimer adatoms are (a) the atom-top site (T), (b) the bond-top site (B) and (c) the hex-center site (H) which is also represented in Fig. 1a. The optimized geometries of these structures are compared to the experimental data of these complexes to understand the bond length and structure evolution of 2D infinite graphene.^24^ Further these structures has been used for metals adsorption. Final optimized geometries of these complexes are given in Fig. 2a and b. The mean bond length of C1–C3 is comparable to the C–C bond value in infinite graphene (1.415 Å).^25^ Two classes of C–C bonds can be distinguished for C1 and C3. The one C–C bond, which is located in the benzenoid rings, has a shorter mean length, whereas the longer bond which connects the benzenoid rings has more of a single-bond character. The values of benzenoid description is mentioned according to the Clar's sextet model.^26^ C2 have a partial zigzag edge, which is unique as the bond lengths are more equalized, but no sextet benzenoid rings can be assigned based on the distinction of bond lengths to C2. It has been observed that the chlorine atoms of C1–C3 induce severe steric hindrance and force the outer benzene rings of the carbon framework to flip up and down in an alternating manner as compared to the parent nanographenes (hydrogen terminated). It has been observed that the non-planarity feature has strongly influenced the C1–C3 packing and the major intermolecular interaction within these NGRs consists of Cl–π and Cl–Cl short contacts without close π–π interactions.

### 3.2 Adsorption energies and geometries

The adsorption energy (*E*_ad_) is defined as:

1 *E*_ad_ = *E*_tot_ − *E*_M_ − *E*_g_

where *E*_tot_ is the total energy of the adsorbed system, *E*_g_ is the energy of the isolated Cl–graphene nanoribbon and *E*_M_ is the energy of an isolated metal/dimer adatom, which is leveled from the metal atom instead of bulk metal. We have also calculated the adatom height for the single metal atom and the dimer atom which is defined as the difference in the *z* coordinates of the adatom and the average of the *z* coordinates of the carbon atoms in C1–C3 Cl NGRs. The distance between the adatom and its nearest carbon atoms (*d*_M–C_) is also calculated. The distortion (Δ*d*) along with the stable adsorption sites, the adsorption geometries, and the adsorption energies for metal adatoms and the dimer atoms adsorbed on these Cl NGRs are reported in Table 1.

**Table 1:** *E*_ad_ is the adsorption energy, *μ* is the magnetic moment, *h* is the distance of the adatom above Cl NGRs, *d*_M–C_ is the distance between the metal adatom and the nearest carbon atoms, *D*_d_ is the maximum deviation in the *z* direction of the C atoms in Cl NGRs from the average positions

Complexes	Bond distance (Å)	Dimer distance (Å)	−*E*_ad_ (eV)		*h* (Å)	*μ*_B_
			GGA	vdW		
C1–Al	2.341		6.81	6.76	2.276	0.730
C1–Au	3.021		0.93	0.72	2.987	0.738
C1–Cu	2.542		1.54	1.46	2.375	0.703
C1–Ald	2.251	3.881	4.77	4.67	2.157	0.718
C1–Aud	3.012	2.592	3.62	3.02	2.971	0.719
C1–Cud	2.382	2.241	4.53	4.37	2.281	0.736
C2–Al	2.451		1.96	1.58	2.386	0.725
C2–Au	3.031		2.77	2.60	2.987	0.730
C2–Cu	2.982		4.51	4.35	2.798	0.730
C2–Ald	2.223	3.192	1.20	1.17	2.133	0.711
C2–Aud	2.682	2.581	2.20	2.16	2.547	0.715
C2–Cud	2.354	2.244	1.89	1.79	2.139	0.731
C3–Al	2.034		74.92	74.83	2.011	0.724
C3–Au	3.018		76.78	76.66	2.997	0.727
C3–Cu	2.632		77.01	76.88	2.576	0.729
C3–Ald	2.532	2.931	77.90	77.61	2.489	0.719
C3–Aud	2.997	2.603	74.64	74.45	2.818	0.681
C3–Cud	2.634	2.251	76.55	76.24	2.459	0.727

It has been observed that the adatoms of coinage metals (Cu, Au) prefer to adsorb on the T site, while the Al adatom tends to strongly adsorb on the H site (as shown in Fig. 2a and b), which is in accordance to the previous results.^55^ The adsorption energy monotonically decreases with the increasing atomic number within adsorption of Cu and Au metals. The trend in adsorption height and M–C distance are also well correlated with the atomic radii of metal adatoms. As the adatom radius increases, its height and M–C distance increases and the distortion of Cl NGRs decreases, which has been observed in all the studied complexes.

In case of C1–metal complexes, the adsorption energies of Al is larger than the adsorption energies of Cu and Au, which clearly reflect the strong chemisorption of Al and Cu adatoms but a weak chemisorption and strong physisorption of Au atoms. The *E*_ad_ values are also calculated using the vdW-DF method. The derivation of adsorption energy with vdW-DF is within 0.17 eV compared with the GGA method under dipole correction. As the relative trend for *E*_ad_ calculation using the vdW-DF method is in accordance with the GGA results, it indicates that the GGA methodology is appropriate for these systems.

In C2–metal complexes, we observed the same decreasing trend of adsorption energy (Al > Cu > Au) for metals, but the adsorption energies of these complexes reflect strong chemisorption. Similar trend in adsorption energy and strong chemisorption has been observed in C3–metal complexes. In C4–Au and C5–Au complexes, we have observed that the bond distances between Au and nanographenes is 2.289 and 2.799 Å respectively. These result indicate that the interface of the gold clusters and nanographenes are stabilized when the metal atoms are just above the carbon atoms which has already been predicted in the previous studies.^31*b*,32*b*^ As the interaction between the metal adatoms affect the electronic properties of Cl NGRs–metal systems, so two metal atoms has also introduced for these Cl NGRs systems. The aim was to investigate the dimer–NGRs interaction as an application towards metallic nanowires and optoelectronics. As the adatom–adatom interaction and the stability of metal dimers has been studied previously,^34–37^ both the parallel and perpendicular position of dimers are considered for the NGRs plane and the final optimized structures are reported in Fig. 2b. It has been observed that C2–Aud, C2–Cud and C3–Cud form perpendicular positions while C1–Aud, C1–Cud and C3–Aud have slightly deviated position.

The formation energy (*E*_FE_) for the dimers is given by the formula:

2 *E*_FE_ = (*E*_Cl NGR_ − *nE*_m_)/*n*

where the *E*_Cl NGR_ is the calculated total energy of a given Cl NGR, *E*_m_ is the energy per atom of a metal dimer, and *n* is the number of atoms in the metal dimers. According to the formula, the NGRs–dimer complexes has the dimer energy of 8.902 eV for Ald, −1.499 eV for Aud and −1.114 eV for Cud, which clearly predict that Al cannot form dimer and it has been predicted by the previous results also.^46^ The Al adatoms has not formed dimers as the formation energies for Al is higher than the formation energies for other coinage metal atoms. So the two adatoms for Al behave as separated atoms for Cl NGRs–Ald complexes. The adsorption of metal and metal dimers has induced a net magnetic dipole moment of 0.73 *μ*_B_ for all the studied complexes.

## 4. Electronic properties

The theoretical and experimental analysis of the C1–C5 show that the electron-withdrawing effect of chlorine lowers the energetic position of the frontier molecular orbitals for all Cl NGRs (C1–C5) by around 1.0 eV, compared to the respective hydrogen-terminated analogues. The calculated energy position of the LUMO in C1–C5 spans from −3.37 eV to −4.01 eV, which is suited for electron injection in n-channel semiconductors.^32^ It also indicate the application of these Cl NGRs as electronic acceptors in devices. The adsorption of metal and metal dimers tunes the band gap within these complexes. Thus these metals can be used as a powerful protocol to decrease the band gap of the Cl NGRs. The HOMO LUMO (HL) gap, Mulliken charge, dipole moment and polarizability of complexes are given in Table 2. The decrease in the optical gap is an effect of the asymmetric stabilization of the frontier molecular orbitals when substituting hydrogen with electron-withdrawing chlorines. The HOMO is having the smaller effect, which is due to the compensation of mesomeric electron donating effect.

**Table 2:** The HOMO–LUMO (HL) gap (eV), Mulliken charges (adsorbed metal and interacted carbon), ground state dipole moment (*D*) and polarizability of the studied complexes

Complexes	HL gap (eV)	Charge (metal)	Charge (carbon)	Dipole moment (*D*)	Polarizability
C1–Al	1.23	0.6413	0.1800	12.74	−157.67
C1–Au	1.50	0.4019	−0.1568	11.65	−166.01
C1–Cu	1.53	0.4596	−0.3588	14.05	−157.43
C1–Ald	2.04	0.4907	−0.0442	4.90	−189.73
C1–Aud	2.00	0.1940	−0.5855	3.88	−184.23
C1–Cud	2.14	0.1345	−0.4021	3.49	−170.42
C2–Al	2.13	0.6331	−0.2906	9.76	−225.41
C2–Au	1.53	0.0043	0.3076	6.73	−209.87
C2–Cu	1.99	0.0043	0.1039	6.62	−213.45
C2–Ald	1.72	0.4783	−0.1641	9.45	−221.84
C2–Aud	2.26	0.0954	−0.0367	6.67	−214.01
C2–Cud	2.34	0.1281	−0.1884	1.10	−197.00
C3–Al	3.01	0.7860	−0.5776	2.56	−234.39
C3–Au	2.71	0.0535	−0.5314	0.50	−237.65
C3–Cu	3.05	0.0138	−0.1889	0.32	−233.01
C3–Ald	2.05	0.4104	−0.1732	2.20	−233.59
C3–Aud	3.05	0.0121	−0.5776	0.80	−240.98
C3–Cud	2.05	0.0138	−0.1889	1.20	−235.74

**Fig. 3:**
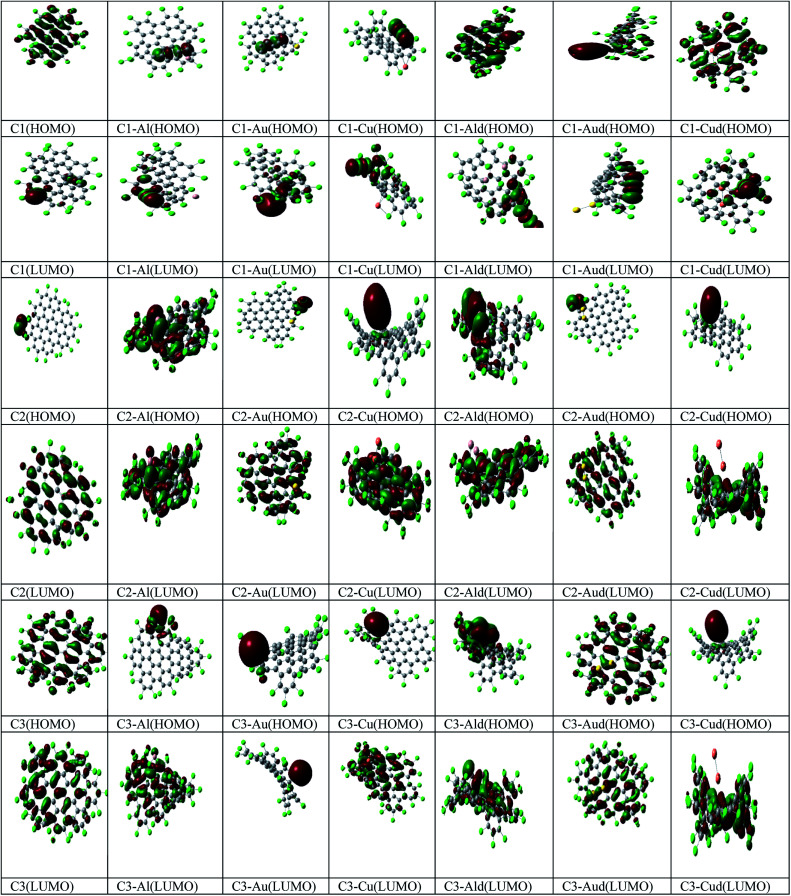
Pictorial representation of the HOMO, LUMO for C1, C2, C3, C1–M, C2–M, C3–M, C1–Md, C2–Md and C3–Md (where M – Al, Au, Cu).

The Mulliken analysis of the charge distribution is used to understand the nature of the interaction between metal adatoms and the Cl NGRs and to evaluate the induced effects on C1, C2 and C3 (Table 2). The trend of charge transfer (Δ*q*) can be understood on the basis of relative electron withdrawing or -donating capability of the metal adatoms. A positive value of Δ*q* means charge transfer from the metal adatom to C1, C2 and C3. The pictorial representation of HOMO and LUMO is also represented in Fig. 3.

In C1–Al complexes, the charges are mostly localized on carbon and metal atoms at HOMO and LUMO, while for C1–Au/Cu complexes, the charges are mostly localized on carbon and chlorine atoms. In C1–Md complexes, the charges are mostly localized on the metal dimer and C1, while the localization of charge is entirely on C1 at LUMO.

Further for C2–Al complexes, the charges are mostly localized on carbon and metal atoms at HOMO and LUMO, while for C2–Au and C2–Cu complexes, the delocalization of charges is observed for coinage metals at HOMO and LUMO. In C2–Md complexes, the charges are mostly localized on the metal dimers (Al, Cu) and C2 at HOMO, while the charges are localized on C2 for all the three C2–Md complexes. For C3–M complexes, the charges are localized mostly on metal at HOMO, while the charges are localized on C3 for C3–Al and C3–Cu complexes. For C3–Md complexes, the charges are localized at metal dimers at HOMO, except for C3–Cud complexes, where the charges are localized at C3 (LUMO).

## 5. Optical properties of edge chlorinated NGR–metal dimers:

**Fig. 4:**
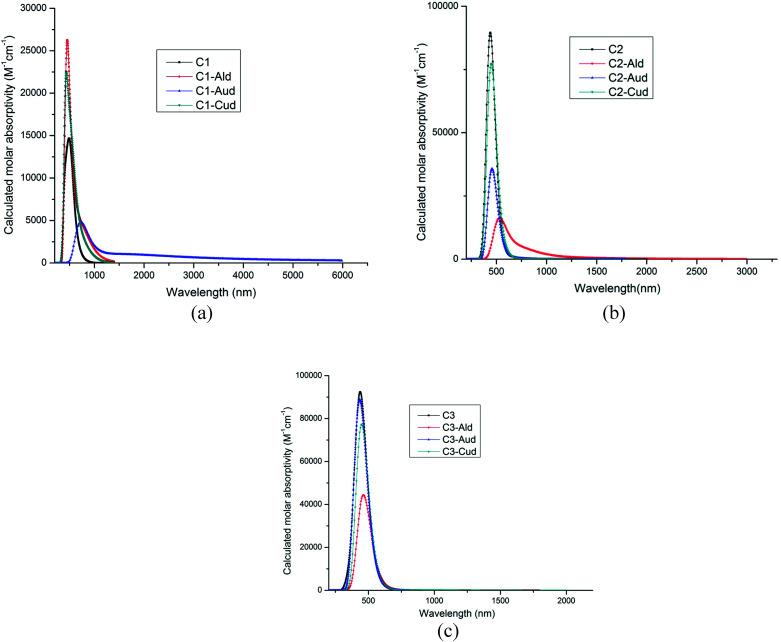
(a) Absorption spectra of C1–Md complexes (M = Al, Au and Cu). (b) Absorption spectra of C2–Md complexes (M = Al, Au and Cu). (c) Absorption spectra of C3–Md complexes (M = Al, Au and Cu).

The calculated wavelength, oscillatory strength, transitions and excited state dipole moment is given in Table 3. As the ultraviolet-visible-near-infrared optical absorption bands of C1–C3 are sharper and exhibit more abundant fine structures than those of alkyl-substituted counterparts,^31^ so the wavelength for the dimer metal atoms are calculated using TDDFT calculations. In all the complexes, the excited state dipole moment showed decreased value as compared to the ground state dipole moment. These results indicate strong configurational mixing associated with near degeneracy of the frontier molecular orbitals. The absorption bands of C1–C3 show a bathochromic shift of 40–50 nm with respect to the hydrogen-terminated compounds.^31^ Edge chlorination can tune the optical property of GNRs as well. This has been seen by the dimer metal atom adsorbed at the C1–C3 surface. The absorption of Cl GNRs is red-shifted by 37 nm. The dimer adsorbed metal–NGR complexes show absorption spectra in visible region (435–723 nm), (Fig. 4a–c), which can utilize these Cl NGRs–Md complexes in the field of optoelectronics.

**Table 3:** The wavelength (nm), oscillatory strength, transitions and excited state dipole moment of the studied complexes by TDDFT calculations

Complexes	Wavelength (nm)	Oscillatory strength (*f*)	Transitions	*μ*_E_ (D)
C1	494.08	0.0526	H−2 → L(61%)	1.03
C1–Ald	454.19	0.0699	H−6 → L(38%)	4.72
C1–Aud	723.13	0.0533	H−1 → L+1(77%)	3.66
C1–Cud	436.18	0.0826	H−1 → L+2(75%)	3.29
C2	438.06	1.0518	H−4 → L+1(49%)	2.38
C2–Ald	549.74	0.0844	H–4 → L(33%)	9.31
C2–Aud	454.36	0.4327	H−6 → L+1(19%)	6.47
C2–Cud	449.47	0.0038	H−7 → L+1(28%)	1.05
C3	436.80	0.6525	H−1 → L(17%)	2.17
C3–Ald	467.01	0.0013	H−2 → L+2(81%)	4.37
C3–Aud	435.57	0.1818	H−2 → L+1(38%)	0.80
C3–Cud	472.59	0.4400	H−5 → L(17%)	1.20

## 6. Thermal conductivity of edge chlorinated NGR–metal dimers:

The heat transport properties of NGRs are considered as an important tool for thermoelectric performance and thermal management. Several studies have been made on the phonon dispersion of GNRs. It has been observed that the thermal conductivity of graphene and graphite is pressure dependent. The electrical conductivity is higher for natural graphite as compared to synthetic graphite at 30 MPa. Although the variation is also density dependent yet the opposite trend is seen in CNTs, synthetic and natural graphene, which show higher electrical conductivity.^47^ As it has already been reported in the previous studies that the thermal conductance of GNRs is independent of ribbon length in the ballistic regime,^48^ so the studies has been carried out at 300 K for Cl NGR–metal dimers. This ballistic assumption is valid for small systems, however, for large-area graphene, diffusive (Umklapp-limited) scattering also plays a significant role in reducing thermal conductivity, as determined theoretically^49^ and experimentally.^50,51^

The thermal conductivity for all nine NGRs–metal dimers complexes has been calculated and it was found that the electrical conductivity for these complexes are enhanced as compared to the conductivity measured for graphene nanoribbons.^45^ It remains interesting to determine if thermal conductance in Cl NGRs–Md complexes follows the same trends as that for energy gap and edge energy, as this will yield further insight on thermal transport mechanisms in Cl NGRs–Md. This may shed some light in understanding the thermoelectric behavior of Cl NGRs–Md complexes, and ultimately be useful for applications in nanoscale electronics.

**Fig. 5:**
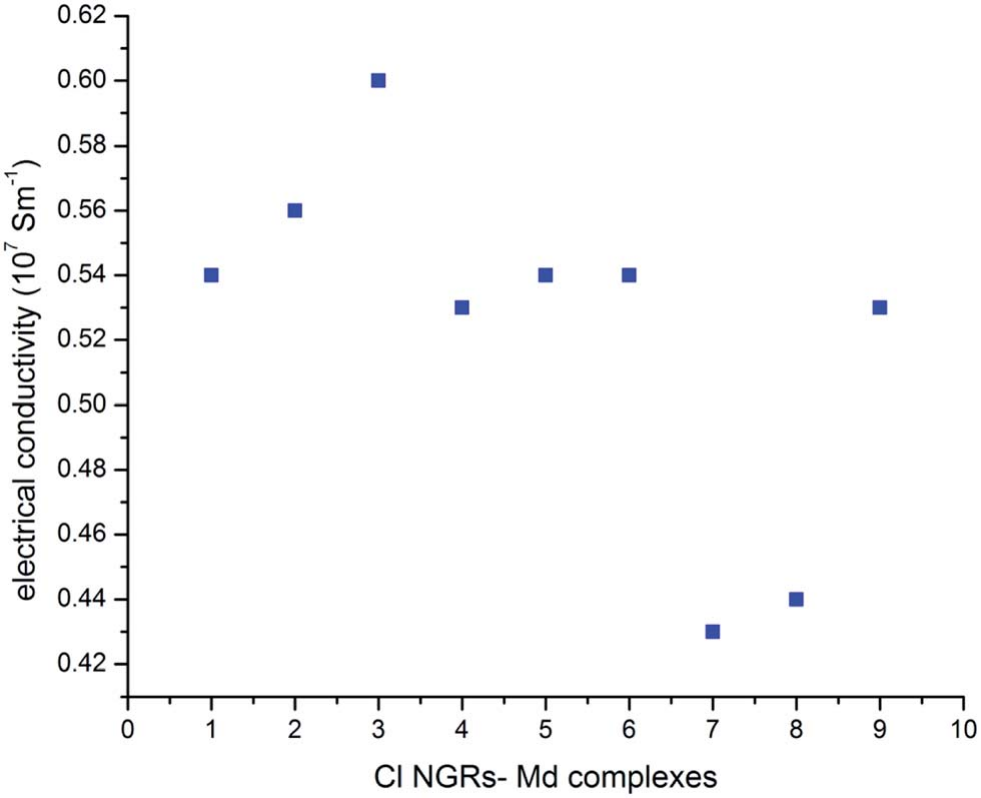
Electrical conductivity (10^7^ S m^−1^) of Cl NGRs–dimer complexes. The number represents (1) C1–Ald, (2) C1–Aud, (3) C1–Cud, (4) C2–Ald, (5) C2–Aud, (6) C2–Ald, (7) C3–Ald, (8) C3–Aud and (9) C3–Cud complexes.

In all the studied complexes, the Cu dimer show higher conductivity as compared to the Au and Al adsorbed metal dimers. This trend is consistent with the HL gap values except for C3–Md complexes, in which the HL gap is higher for C3–Aud complexes rather than C3–Cud complexes (Fig. 5). As we indicated that these functionalized materials have a potential application in thermo-electric fields, interestingly the similar devices have been designed recently.^52,53^

## 7. Conclusions

First principle analysis has been carried out on the electronic, magnetic, optical and transport properties of the different metal conductive adatoms (Al, Cu and Au) adsorbed on (C1–C3) Cl NGRs. On the basis of DFT calculations, it was found that all the studied complexes show strong chemical bonding with Cl-NGRs, while the adsorption of Au on C1 is between weak chemisorption and strong physisorption. These metal–Cl NGRs systems have net magnetic moment of 0.73 *μ*_B_. The TDDFT calculations for metal dimers–Cl NGRs systems show absorption in the visible region. It is demonstrated that these complexes can serve as an efficient approach to modulate the properties of nanographenes. This strategy can lead to design and tune the Cl-NGRs properties with the single and dimer adatoms required by most practical technologies. The results mentioned above may be valuable in the design of metal adsorbed-Cl NGRs based electronic devices, and show promise for metallic nanowire applications in the future.

## Conflicts of interest

There are no conflicts to declare.

## Supplementary Material

RA-008-C8RA02032A-s001
