# Supplementary material for: Theoretical studies of optoelectronic, magnetization and heat transport properties of conductive metal adatoms adsorbed on edge chlorinated nanographenes
Source: RSC Adv. 2018 May 15;8(32):17723–31. doi: 10.1039/c8ra02032a (PMC9080487; doi:10.1039/c8ra02032a)
Supplement: RA-008-C8RA02032A-s001 [file RA-008-C8RA02032A-s001.pdf]

Electronic Supplementary Material (ESI) for RSC Advances.
This journal is © The Royal Society of Chemistry 2018

Supplementary Information

Title: Theoretical studies of optoelectronic, magnetization and heat transport properties of conductive metal adatoms adsorbed on edge chlorinated nanographenes

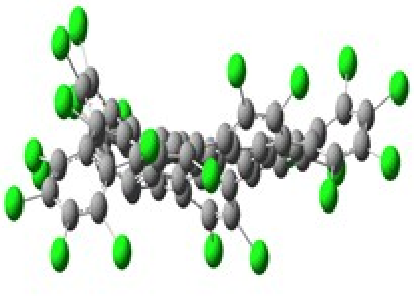

$C4(C_{60}Cl_{24})$

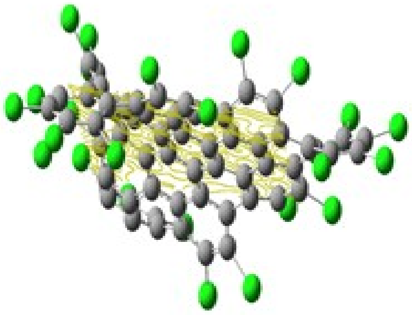

C4 (ESP -2189.11au)

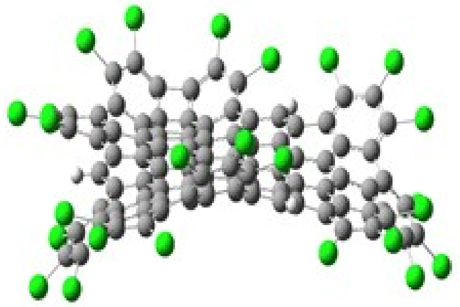

$C5(C_{96}Cl_{27}H_3)$

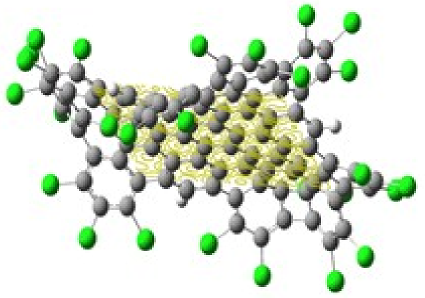

C5 (ESP -3146.94 au)

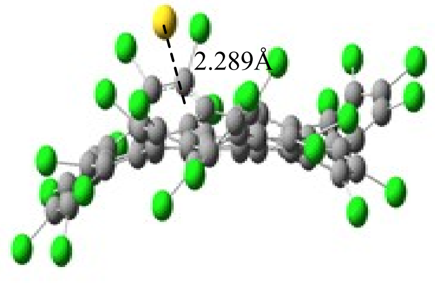

C4-Au

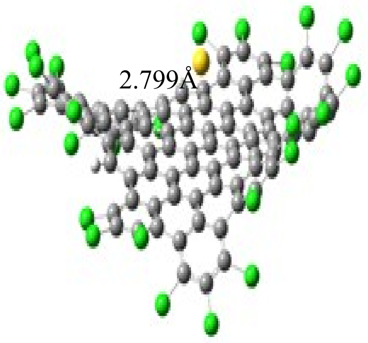

C5-Au

Supplementary Figure 1.The optimized structures for C4 ($C_{60}Cl_{24}$), C5 ($C_{96}Cl_{27}H_3$), C4-Au and C5-Au complexes along with the ESP potential surface for the isolated monomers.

Complexes	HL gap (eV)	Dipole Moment(D)	polarizability	ESP (au)	Energy (ev)	Energy (ev)vdW	Bond distance (Å)
C4	2.09	1.933	-243.11	-2189.11	-587.35	-	
C5	2.31	0.932	-244.78	-3146.94	-937.96	-	
C4-Au	2.03	1.311	-253.77	-	-591.22	-593.23	2.289
C5-Au	2.01	1.342	-251.33	-	-941.11	-944.32	2.799

Supplementary Table 1. The HOMO-LUMO (HL) gap (eV), ground state dipole moment(D), polarizability, ESP values (monomers) and energy of the studied complexes. The eigen values and eigenstates of the Kohn-Sham Hamiltonian have been calculated at Γ point only of a cubic cell of side (C4-22.077 Å, C5-27.360Å, C4-Au-25.399Å, C5-Au-30.077Å). All the other computational details are given in the manuscript.

Complexes	Wavelength (nm)	Oscillatory strength (*f*)	Transitions	μ_E(D)
C4	462.52	0.2847	H→L+3 (50%)	4.97
C5	645.81	0.0001	H→L (80%)	4.72
C4-Au	444.52	0.0022	H→L+2(55%)	3.95
C5-Au	532.11	0.2132	H→L+3(49%)	3.11

Supplementary Table 2. The Wavelength(nm), Oscillatory strength, transitions and excited state dipole moment of the studied complexes by TDDFT calculations
